# Hepatitis C Virus Genotype Distribution in Istanbul, Türkiye: Temporal Trends and Demographic Patterns

**DOI:** 10.3390/v18101073

**Published:** 2026-09-29

**Authors:** Leyla Genç, Hakan Karaca, Mehmet Emin Bulut

**Affiliations:** Department of Medical Microbiology, University of Health Sciences, Şişli Hamidiye Etfal Training and Research Hospital, Istanbul 34475, Türkiye; hakankkrc@gmail.com (H.K.); eminbulut212@gmail.com (M.E.B.)

**Keywords:** hepatitis C virus, HCV genotype, genotype distribution, genotype 3, molecular epidemiology, temporal trends, Türkiye

## Abstract

Hepatitis C virus (HCV) genotype distribution varies across geographic regions and populations and may change over time. This study examined the current distribution and temporal trends of HCV genotypes, and their patterns by demographic characteristics and nationality, at a tertiary care hospital in Istanbul, Türkiye. We retrospectively evaluated 188 patients who underwent HCV genotyping between June 2019 and December 2024. Genotype distribution was assessed according to age, sex, and year, while temporal trends were analyzed using the Cochran–Armitage trend test and binomial logistic regression. GT1b was the predominant subtype (54.8%), followed by GT3 (19.1%), GT1a (10.1%), and GT4 (5.9%); mixed-genotype results were detected in 5.9% of cases. Genotype distribution differed significantly by age group and sex (Monte Carlo *p* = 0.044 and *p* = 0.002, respectively), but not by year (*p* = 0.128). After Holm adjustment, GT1a was more frequent among males, whereas GT1b was more frequent among females. No significant linear temporal trends were observed for GT1a, GT1b, GT3, GT4, or mixed-genotype results. Genotype patterns also varied descriptively across nationality groups. These findings underscore the importance of up-to-date regional data and continued genotype surveillance for monitoring changes in HCV epidemiology.

## 1. Introduction

Hepatitis C virus (HCV) infection remains a major global health concern due to its potential to cause chronic liver disease, cirrhosis, and hepatocellular carcinoma [1]. An estimated 47 million people worldwide are living with chronic HCV infection, with approximately 0.9 million new infections occurring annually [1].

HCV exhibits substantial genetic diversity and is currently classified into eight genotypes and more than 90 subtypes [2,3]. HCV genotypes differ by approximately 30–35% at the nucleotide sequence level, whereas genetic divergence between subtypes exceeds 15% [3]. HCV genotype distribution varies considerably across geographic regions [4]. GT1 and GT3 are the two most prevalent genotypes worldwide, accounting for approximately 46% and 30% of infections, respectively [4]. GT1a and GT1b are commonly found in Europe, North America, and Japan, whereas GT3 is particularly prevalent in parts of Asia and is also frequently reported among people who inject drugs [4,5]. GT4 is predominantly found in North Africa and the Middle East [4]. In Türkiye, GT1b has historically been the predominant HCV genotype [6,7].

Direct-acting antivirals (DAAs) targeting the HCV NS3 protease, NS5A protein, or NS5B RNA-dependent RNA polymerase have substantially transformed the treatment of chronic HCV infection [8]. The introduction of highly effective pangenotypic DAA regimens has reduced the role of genotype determination in treatment selection [8]. Nevertheless, genotype information may remain relevant in certain clinical settings due to virological characteristics that may influence treatment response in specific genotypes and subtypes [8]. Characterization of circulating HCV genotypes also remains valuable for molecular epidemiology and surveillance [4,7]. Monitoring genotype distribution over time may help identify changes in circulating viral populations as well as regional and demographic differences [7]. Although numerous studies have investigated HCV genotype distribution in Türkiye, ongoing demographic changes and potential temporal shifts in genotype distribution highlight the importance of up-to-date regional data.

This study aimed to determine the current distribution and temporal trends of HCV genotypes among patients evaluated at a large tertiary training and research hospital in Istanbul, and to examine the associations between genotype distribution and age, sex, and year, as well as its variation across nationality groups.

## 2. Materials and Methods

### 2.1. Study Design and Data Collection

This retrospective study included 188 patients who tested positive for both anti-HCV antibodies and HCV RNA and underwent HCV genotyping at the medical microbiology laboratory of a tertiary training and research hospital in Istanbul between June 2019 and December 2024. Data on age, sex, nationality, and clinical diagnosis were retrieved from the hospital information management system.

### 2.2. Laboratory Methods

Quantitative HCV RNA testing was performed using real-time reverse transcription polymerase chain reaction (RT-PCR) on the Cobas 6800 System (Roche Diagnostics GmbH, Mannheim, Germany) from June 2019 to May 2024 and on the ELITe BeGenius platform (ELITechGroup S.p.A., Torino, Italy) from June 2024 to December 2024. The change in the HCV RNA quantification platform reflected a change in the routine laboratory workflow during the study period. HCV RNA quantification was part of the routine diagnostic evaluation and was not included as a quantitative variable in the comparative or temporal analyses of genotype distribution. HCV genotyping was performed using the Abbott RealTime HCV Genotype II assay (Abbott Molecular Inc., Des Plaines, IL, USA) from June 2019 to December 2020 and the Bosphore HCV Genotyping Kit v5 (Anatolia Geneworks, Istanbul, Türkiye) with the Montania 4896 Real-Time PCR System from January 2021 to December 2024. The Abbott RealTime HCV Genotype II assay targets the 5′ untranslated region (5′UTR) to determine the genotype and the NS5B region to differentiate between GT1a and GT1b. The Bosphore HCV Genotyping Kit v5 targets the NS5B region and detects HCV genotypes using fluorescence-based detection. The change in genotyping platforms during the study period reflected a transition in the routine laboratory testing system.

### 2.3. Statistical Analysis

Statistical analyses were performed using SPSS version 15.0 for Windows and Python 3.13.5 (NumPy 2.3.5, SciPy 1.17.0, and statsmodels 0.14.6). Categorical variables were summarized as numbers and percentages, whereas continuous variables were presented as means, standard deviations, and minimum and maximum values. All 188 patients and eight mutually exclusive observed genotype categories were included in overall comparisons by study year, age group, and sex. Because of sparse cells, Pearson chi-square statistics were evaluated using fixed-margin conditional Monte Carlo inference (1,000,000 tables per test). Pairwise age-group and year comparisons were performed using the same procedure, whereas genotype-specific sex comparisons were performed using two-sided Fisher’s exact tests. Holm correction for multiple comparisons was applied separately across all 21 age-group pairwise comparisons, eight genotype-specific sex comparisons, and 15 pairwise year comparisons. Overall *p*-values were unadjusted, whereas Holm-adjusted *p*-values were used for pairwise comparisons. Nationality distributions were evaluated descriptively.

GT1 (non-1a/1b) was defined as GT1 results not further subtyped as GT1a or GT1b. Temporal trends were assessed for GT1a, GT1b, GT1 (non-1a/1b), GT3, GT4, and mixed-genotype results using two-sided Cochran–Armitage tests for trend with equally spaced year scores (0–5 for 2019–2024). Unadjusted binary logistic regression models were additionally fitted for each of these genotype categories, with the respective genotype category coded as the outcome (1 = target category; 0 = all other categories) and calendar year, centered at 2019, entered as the sole continuous predictor. Thus, no categorical reference year was used, and the resulting odds ratios (ORs) represent the change in odds associated with a one-year increase in calendar year. GT2 and GT5 were not analyzed separately because each was detected in only one patient; however, these patients were retained in the annual denominators and comparison groups. Nominal *p*-values and 95% Wald confidence intervals (CIs) were reported, and Holm correction was applied across the six genotype categories separately for the Cochran–Armitage trend tests and logistic regression Wald tests. A two-sided *p*-value <0.05 was considered statistically significant.

### 2.4. Ethics Approval

The study was conducted in accordance with the Declaration of Helsinki and approved by the Clinical Research Ethics Committee of Şişli Hamidiye Etfal Training and Research Hospital Health Application and Research Center (approval no. 4843; 22 April 2025).

## 3. Results

### 3.1. Patient Characteristics and Overall HCV Genotype Distribution

A total of 188 patients were included in the study; 98 (52.1%) were female, and 90 (47.9%) were male. Patient ages ranged from 4 to 94 years, with a mean age of 54.6 ± 17.6 years. Of the patients, 50.0% were followed in the Infectious Diseases outpatient clinic, 29.3% in the Gastroenterology outpatient clinic, and 20.7% in other outpatient clinics or hospital units. Among the 166 patients for whom clinical diagnosis data were available, 97.6% had chronic hepatitis C and 2.4% had acute hepatitis C.

GT1 was the most frequently detected genotype (68.1%; *n* = 128), followed by GT3 (19.1%; *n* = 36), GT4 (5.9%; *n* = 11), and GT2 and GT5 (0.5% each; *n* = 1 each). GT1b was the predominant subtype, detected in 54.8% of all cases (*n* = 103), while GT1a was detected in 10.1% (*n* = 19), and GT1 not further subtyped as GT1a or GT1b was detected in 3.2% (*n* = 6). All 188 patients included in the study were successfully genotyped. A single HCV genotype was detected in 177 patients, whereas mixed-genotype results were obtained in the remaining 11 patients (5.9%). In mixed-genotype results, GT1 denotes genotype 1 that was not further subtyped as GT1a or GT1b. Three patients had a GT1/GT1b combination, while the remaining combinations were GT1/GT1a, GT1/GT1a/GT2, GT1a/GT3, GT1b/GT4, GT1b/GT3, GT2/GT3, GT3/GT4, and GT3/GT6 (*n* = 1 each).

### 3.2. Temporal Distribution and Trends of HCV Genotypes

In the complete eight-category table, genotype distribution did not differ significantly across study years (χ^2^ = 44.562, df = 35; conditional Monte Carlo *p* = 0.128). GT1b was the most frequently detected subtype throughout the study period, whereas GT3, which was not detected in 2019, accounted for 17–27% of annual results between 2020 and 2024. Mixed-genotype results were detected in all years except 2023. Of the 15 exploratory pairwise comparisons between study years, only the comparison between 2019 and 2021 remained significant after Holm adjustment (adjusted *p* = 0.007); however, this isolated pairwise difference does not indicate an overall or continuous temporal trend. The distribution of HCV genotypes by year is presented in Table 1; the annual proportional distribution of genotypes is shown in Figure 1, and the pairwise comparisons are provided in Supplementary Table S1.

The Cochran–Armitage test for trend showed no significant linear temporal trends for GT1a, GT1b, GT3, GT4, or mixed-genotype results (all nominal *p* > 0.05). In contrast, GT1 (non-1a/1b) showed a significant decreasing linear trend over the study period (Z = −2.864; nominal *p* = 0.004; Holm-adjusted *p* = 0.025). Unadjusted binary logistic regression also showed a significant negative association with year for this category, with an annual OR of 0.092 (95% CI 0.017–0.491; nominal Wald *p* = 0.005; Holm-adjusted *p* = 0.031), whereas no significant temporal associations were observed for the other genotype categories (all nominal *p* > 0.05). This category comprised six patients, four in 2019 and two in 2020. Full trend and regression results are provided in Supplementary Table S2.

### 3.3. Genotype Distribution According to Demographic Characteristics and Nationality

Genotype distribution differed significantly by sex (χ^2^ = 20.295, df = 7; conditional Monte Carlo *p* = 0.002). Although GT1b was predominant in both sexes, it was more frequent in females than in males (66% vs. 42%), whereas GT1a was more frequent in males than in females (18% vs. 3%). In genotype-specific comparisons, these sex differences remained significant after Holm adjustment (adjusted *p* = 0.009 for both). GT3 was numerically more frequent in males than in females (24% vs. 14%), but the difference was not statistically significant (adjusted *p* = 0.572). The distribution of HCV genotypes by sex is shown in Figure 2, and the full genotype-specific comparisons are provided in Supplementary Table S1.

Patients were most frequently represented in the 41–50-year-old age group (24.5%; *n* = 46), followed by the 51–60 and ≥71-year-old age groups (20.2%; *n* = 38 each). Genotype distribution differed significantly across age groups (χ^2^ = 69.915, df = 42; conditional Monte Carlo *p* = 0.044). After Holm adjustment across 21 pairwise age-group comparisons, the genotype distribution in patients aged ≥71 years differed significantly from that in the 31–40, 41–50, 51–60, and 61–70-year-old age groups (adjusted *p* = 0.001, *p* < 0.001, *p* = 0.004, and *p* = 0.010, respectively); no other pairwise age-group comparison was significant. These comparisons reflect differences in the overall genotype distribution rather than individual genotype proportions. GT1b accounted for 84% of results in the ≥71-year-old age group, whereas GT3 was numerically most frequent in the 31–40 (37%) and 41–50 (30%) age groups. Detailed age-specific distributions are presented in Table 2, and pairwise comparisons are provided in Supplementary Table S1.

Nationality data were available for 187 patients, of whom 121 (64.7%) were Turkish citizens and 66 (35.3%) were foreign nationals. Among foreign nationals, patients from Turkmenistan constituted the largest group (47%), followed by those from Syria (17%) and Uzbekistan (12%). GT1b was predominant among patients from Turkmenistan (77%) and Uzbekistan (63%), whereas GT4 was the most frequently detected genotype among patients from Syria (45%). The distribution of HCV genotypes by nationality among foreign nationals is presented in Table 3.

## 4. Discussion

In our study, GT1b was the most frequently detected subtype, accounting for 54.8% of cases, with GT3 accounting for 19.1%. Mixed-genotype results were observed in 5.9% of cases. Genotype distribution did not differ significantly across the study years, and no significant linear temporal trends were identified for the major genotypes. Genotype distribution differed significantly by age and sex; GT1b was more frequent among females and accounted for a high proportion of cases in the oldest age group, whereas GT1a was more frequent among males. Among foreign nationals, different genotype distribution patterns were observed across nationality groups.

Although GT1 remains the most prevalent HCV genotype worldwide, its distribution varies considerably across geographic regions [4]. GT1b has historically been the predominant subtype in Türkiye and has been reported at high proportions across different regions of the country [6,7,9]. Consistent with this distribution in Türkiye, GT1b was also the most frequently detected subtype in our study, accounting for 54.8% of cases. However, this proportion was lower than the 67.8% previously reported at our center [6]. Despite this difference between the two study periods, GT1b remained the most frequently detected subtype throughout the current study period and showed no significant linear temporal trend. A recent meta-analysis from Türkiye similarly reported that GT1 remained predominant, although its proportion decreased after 2011 [7].

The proportion of GT3 was 19.1% in the present study, whereas a previous study from our center reported 8.8% [6]. In Türkiye, the proportion of GT3 has been reported to increase significantly after 2011 [7], while recent studies from Istanbul have reported rates of approximately 14–16% [10,11,12]. Similar findings have also been reported from Greece; in a recent study, GT3a was the most frequently detected subtype, accounting for 50.2% of cases [13]. Although high sustained virological response (SVR) rates are achieved in GT3 infection with pangenotypic DAA regimens, lower SVR rates have been reported in certain clinical settings, particularly among patients with cirrhosis or prior treatment experience [8,14]. Furthermore, treatment selection and duration for GT3 infection may vary according to clinical and virological characteristics, indicating that genotype information remains relevant in certain clinical settings [8]. Therefore, monitoring the regional distribution of GT3 remains important for both epidemiological surveillance and clinical assessment.

Temporal trend analysis showed no significant linear trends for GT1a, GT1b, GT3, GT4, or mixed-genotype results. Only GT1 results not further subtyped as GT1a or GT1b showed a significant decreasing trend. However, this category included only six patients, with cases confined to 2019 (*n* = 4) and 2020 (*n* = 2). Therefore, the observed decreasing trend should be interpreted cautiously from an epidemiological perspective. Although the proportion of GT3 was higher than that reported in our previous center-based study, no significant increasing linear trend was observed during the current study period. In contrast, an eight-year study conducted in Central Anatolia reported that the proportion of GT3 increased from 0% in 2014 to 11.5% in 2021, with a significant increasing trend (Z = 3.572; *p* = 0.0004) [15]. These findings suggest that temporal trends in HCV genotype distribution may vary across regions and populations.

Descriptive differences in HCV genotype distribution were observed across nationality groups among foreign nationals. GT1b was predominant among patients from Turkmenistan and Uzbekistan, whereas GT4 was the most frequently detected genotype among patients from Syria. These findings are consistent with previous reports showing geographic variation in HCV genotype distribution [4]. A study conducted in Central Anatolia reported significant differences in HCV genotype distribution between Turkish citizens and refugees; GT1b was more frequent among Turkish citizens, whereas GT1a, GT3, and GT4 were more frequent among refugees [15]. In a study from Southeastern Anatolia, the proportion of GT1 was reported to decrease over time, while that of GT4 increased; these changes were discussed in relation to the potential impact of migration on genotype distribution [16]. However, these nationality-related findings should be interpreted as descriptive and exploratory. Foreign patients were identified throughout the study period, and the composition of this population may have varied over time. Moreover, nationality does not indicate the country in which HCV infection was acquired, and detailed migration histories were not available. Therefore, the observed differences cannot be used to infer specific epidemiological associations between nationality and HCV genotype.

In our study, genotype distribution differed significantly by both sex and age group. GT1a was more frequent in males, whereas GT1b was more frequent in females; these sex differences remained significant after adjustment for multiple comparisons. Although GT3 was numerically more frequent in males, this difference was not statistically significant after Holm adjustment. The highest proportions of GT3 were observed in the 31–40- and 41–50-year-old age groups, whereas GT1b accounted for 84% of cases in the ≥71-year-old age group. Data from Türkiye support these findings, showing that GT1/GT1b is more frequently observed among females and older individuals, whereas GT3 is more common among males and younger individuals [7,15]. Similarly, a study from neighboring Greece reported that GT3a was particularly frequent among younger and male patients [13]. However, the lack of data on individual risk factors and routes of transmission limits our ability to assess the potential reasons for the observed differences by age and sex.

GT2 and GT5 were each detected in only 0.5% of cases, consistent with the low proportions reported in previous studies from Türkiye [9,16,17]. Mixed-genotype results were detected in 5.9% of cases. Previous studies from Türkiye have reported mixed-genotype results at rates of 1.1–2.6% [9,18], while a previous study from our center reported a rate of 2.9% [6]. However, because no significant temporal trend was observed for mixed-genotype results in the present study, the higher proportion compared with previous studies should not be interpreted as evidence of an increase over time. When interpreting mixed-genotype results, the ability of the genotyping method to detect the genotype present at a lower proportion should also be taken into consideration [19].

Although the widespread use of pangenotypic direct-acting antiviral regimens has reduced the role of routine genotyping in treatment selection, HCV genotyping remains valuable from an epidemiological perspective [2,8]. Monitoring genotype distribution may help identify changes in circulating viral populations as well as regional and demographic differences [20]. In this context, continued genotype surveillance may provide epidemiological information complementary to general HCV surveillance and contribute to monitoring progress toward HCV elimination [21,22].

The strengths of this study include the opportunity to compare the present findings with previously reported genotype data from the same center and to evaluate temporal trends alongside demographic characteristics and nationality. In addition, the tertiary care setting of our center, which serves patients of diverse nationalities, allowed genotype distribution to be evaluated in a heterogeneous patient population. Nevertheless, the retrospective, single-center design may limit the generalizability of the findings, and detailed data on individual risk factors, routes of transmission, and treatment outcomes were not available. Therefore, the present study primarily provides a descriptive epidemiological overview of HCV genotype distribution, and the underlying factors associated with the observed genotype patterns across demographic groups could not be evaluated. Two different commercial genotyping platforms were used sequentially during the study period due to a change in the routine laboratory workflow. Furthermore, HCV genotyping was based on commercial real-time PCR assays, and sequence-based characterization was not performed. Consequently, sequence-level genetic diversity and phylogenetic relationships among the circulating HCV strains could not be evaluated. Despite these limitations, our study provides up-to-date epidemiological data on HCV genotype distribution in a diverse patient population in Istanbul.

## 5. Conclusions

GT1b remained the predominant HCV subtype at our center, while GT3 was detected at a higher proportion than in our previous center-based data. Nevertheless, no significant linear temporal trends were observed for the major genotypes, including GT3, during the study period. Variation in genotype distribution by age and sex highlights the importance of up-to-date regional data in evaluating HCV genotype epidemiology. Continued genotype surveillance may provide valuable information for monitoring changes in HCV epidemiology.

## Figures and Tables

**Figure 1:**
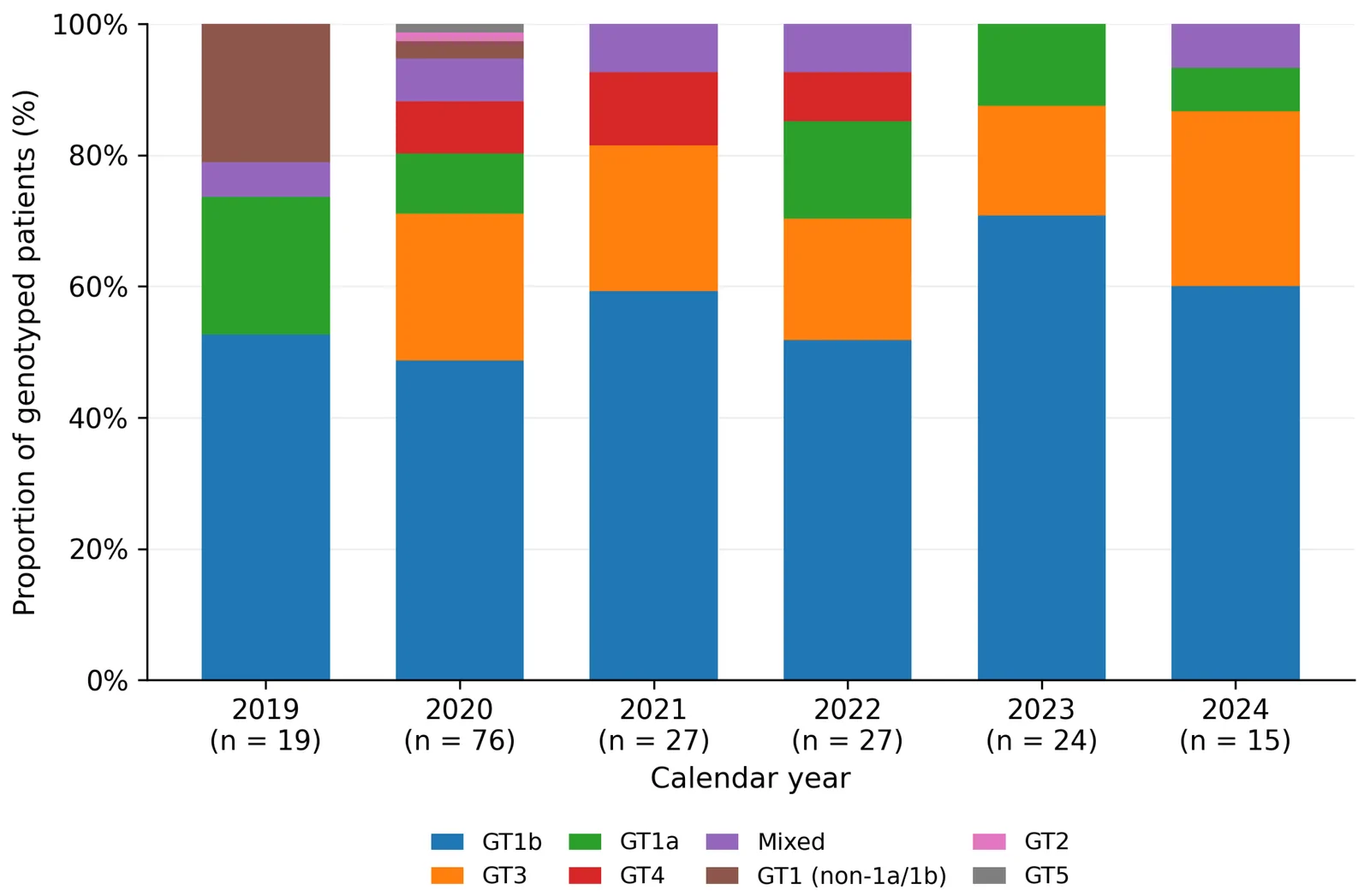
Annual proportional distribution of HCV genotypes, 2019–2024. Each bar represents the genotype distribution among patients genotyped in the corresponding year; annual sample sizes are shown below the bars. GT1 (non-1a/1b) refers to GT1 results not further subtyped as GT1a or GT1b. Data for 2019 represent the available part-year study period.

**Figure 2:**
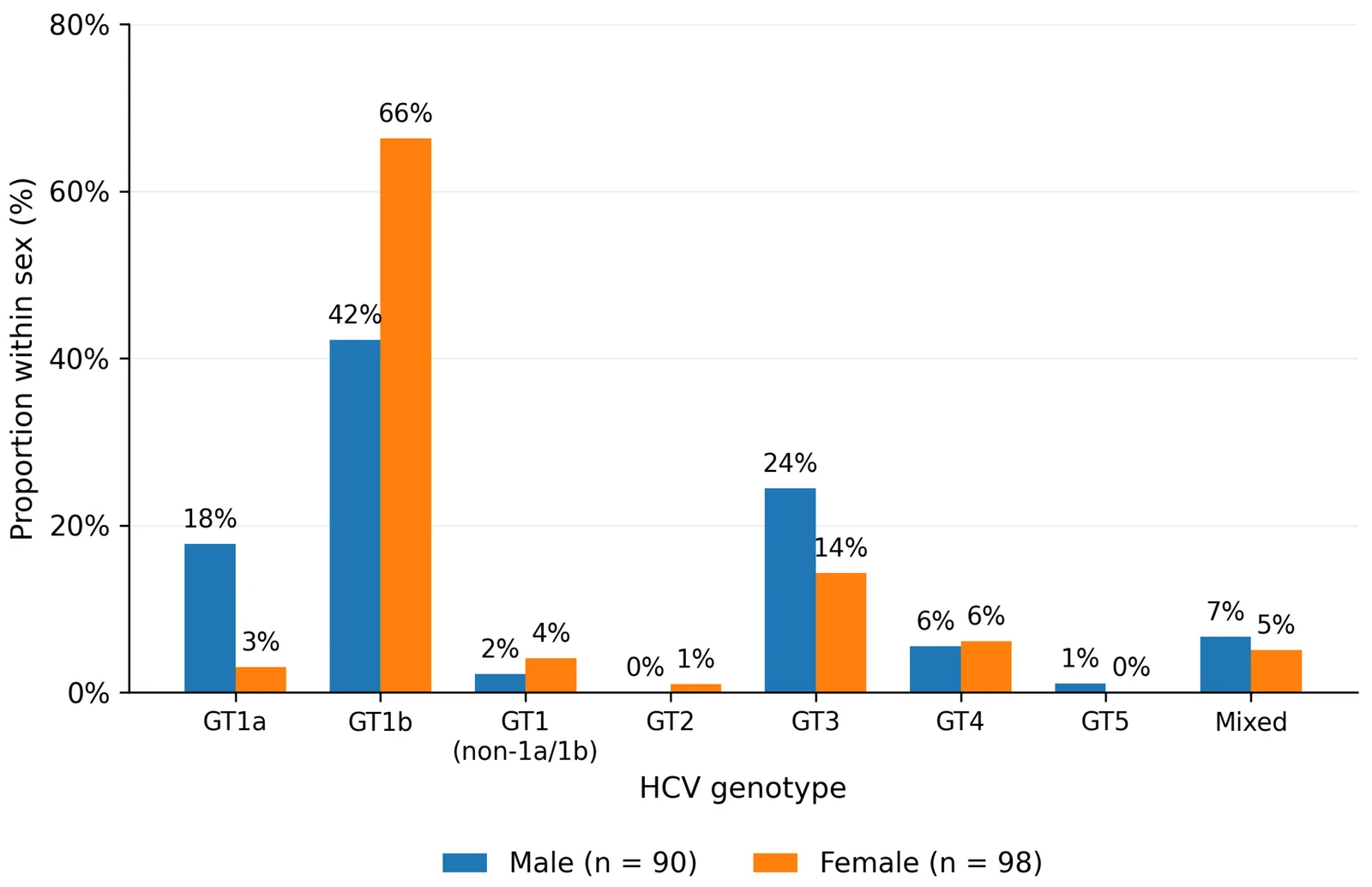
Distribution of HCV genotypes by sex. Percentages represent genotype distributions within each sex. GT1 (non-1a/1b) refers to GT1 results not further subtyped as GT1a or GT1b.

**Table 1:** Distribution of HCV genotypes by study year.

Year	Patients, *n* (%) *								
		Genotype, *n* (%) **							
		1a	1b	1 (Non-1a/1b) ***	2	3	4	5	Mixed
2019	19 (10.1)	4 (21)	10 (53)	4 (21)	–	–	–	–	1 (5)
2020	76 (40.4)	7 (9)	37 (49)	2 (3)	1 (1)	17 (22)	6 (8)	1 (1)	5 (7)
2021	27 (14.4)	–	16 (59)	–	–	6 (22)	3 (11)	–	2 (7)
2022	27 (14.4)	4 (15)	14 (52)	–	–	5 (19)	2 (7)	–	2 (7)
2023	24 (12.8)	3 (13)	17 (71)	–	–	4 (17)	–	–	–
2024	15 (8.0)	1 (7)	9 (60)	–	–	4 (27)	–	–	1 (7)
Total	188 (100.0)	19 (10.1)	103 (54.8)	6 (3.2)	1 (0.5)	36 (19.1)	11 (5.9)	1 (0.5)	11 (5.9)

* Percentages indicate the distribution of patients across study years (*n* = 188). ** Percentages indicate genotype distribution within each year; the total row is based on *n* = 188. *** GT1 (non-1a/1b) refers to GT1 results that were not further subtyped as GT1a or GT1b.

**Table 2:** Distribution of HCV genotypes by age group.

Age Group	Patients, *n* (%) *								
		Genotype, *n* (%) **							
		1a	1b	1 (Non-1a/1b) ***	2	3	4	5	Mixed
0–20	4 (2.1)	1 (25)	1 (25)	–	–	–	1 (25)	–	1 (25)
21–30	13 (6.9)	–	8 (62)	–	–	3 (23)	2 (15)	–	–
31–40	19 (10.1)	2 (11)	9 (47)	–	–	7 (37)	–	–	1 (5)
41–50	46 (24.5)	6 (13)	20 (43)	4 (9)	–	14 (30)	2 (4)	–	–
51–60	38 (20.2)	3 (8)	17 (45)	2 (5)	–	9 (24)	2 (5)	–	5 (13)
61–70	30 (16.0)	7 (23)	16 (53)	–	–	3 (10)	2 (7)	1 (3)	1 (3)
≥71	38 (20.2)	–	32 (84)	–	1 (3)	–	2 (5)	–	3 (8)
Total	188 (100.0)	19 (10.1)	103 (54.8)	6 (3.2)	1 (0.5)	36 (19.1)	11 (5.9)	1 (0.5)	11 (5.9)

* Percentages indicate the distribution of patients across age groups (*n* = 188). ** Percentages indicate genotype distribution within each age group; the total row is based on *n* = 188. *** GT1 (non-1a/1b) refers to GT1 results that were not further subtyped as GT1a or GT1b.

**Table 3:** Distribution of HCV genotypes by nationality among foreign nationals.

Nationality	Patients, *n* (%) *								
		Genotype, *n* (%) **							
		1a	1b	1 (Non-1a/1b) ***	2	3	4	5	Mixed
Turkmenistan	31 (47)	–	24 (77)	2 (6)	–	5 (16)	–	–	–
Syria	11 (17)	4 (36)	1 (9)	–	–	–	5 (45)	1 (9)	–
Uzbekistan	8 (12)	–	5 (63)	1 (13)	–	1 (13)	–	–	1 (13)
Others ****	16 (24)	1 (6)	6 (38)	–	–	7 (44)	–	–	2 (13)
Total	66 (100)	5 (8)	36 (55)	3 (5)	–	13 (20)	5 (8)	1 (2)	3 (5)

* Percentages indicate the distribution of foreign nationals across nationality groups (*n* = 66). ** Percentages indicate genotype distribution within each nationality group; the total row is based on *n* = 66. *** GT1 (non-1a/1b) refers to GT1 results that were not further subtyped as GT1a or GT1b. **** Other nationalities included Azerbaijan (*n* = 3), China (*n* = 1), Georgia (*n* = 2), Kyrgyzstan (*n* = 2), Moldova (*n* = 2), Pakistan (*n* = 1), Russia (*n* = 1), Thailand (*n* = 1), and Ukraine (*n* = 3).

## Data Availability

The data supporting the findings of this study are not publicly available due to privacy and confidentiality restrictions related to patient information.

## Supplementary Materials

The following supporting information can be downloaded at: https://www.mdpi.com/article/10.3390/v18101073/s1, Table S1: Pairwise comparisons of HCV genotype distributions (Table S1a: Pairwise comparisons between age groups; Table S1b: Genotype-specific comparisons by sex; Table S1c: Pairwise comparisons between study years); Table S2: Genotype-specific temporal trend and logistic regression analyses (N = 188).

## Abbreviations

The following abbreviations are used in this manuscript:

HCV	Hepatitis C virus
GT	Genotype
DAA	Direct-acting antiviral
SVR	Sustained virological response
