# Supplementary material for: Hepatitis C Virus Genotype Distribution in Istanbul, Türkiye: Temporal Trends and Demographic Patterns
Source: Viruses. 2026 Sep 29;18(10):1073. doi: 10.3390/v18101073 (PMC13709762; doi:10.3390/v18101073)
Supplement: Supplementary file 1 [file viruses-18-01073-s001.zip › viruses-4558358-supplementary.pdf]

Supplementary Materials

Supplementary Table S1. Pairwise comparisons of HCV genotype distributions

Table S1a. Pairwise comparisons between age groups

Group 1	Group 2	n1	n2	Raw p (MC)	Holm-adjusted p
0–20	21–30	4	13	0.120	1.000
0–20	31–40	4	19	0.069	0.908
0–20	41–50	4	46	0.031	0.459
0–20	51–60	4	38	0.458	1.000
0–20	61–70	4	30	0.360	1.000
0–20	≥71	4	38	0.025	0.400
21–30	31–40	13	19	0.232	1.000
21–30	41–50	13	46	0.220	1.000
21–30	51–60	13	38	0.389	1.000
21–30	61–70	13	30	0.336	1.000
21–30	≥71	13	38	0.015	0.257
31–40	41–50	19	46	0.424	1.000
31–40	51–60	19	38	0.638	1.000
31–40	61–70	19	30	0.186	1.000
31–40	≥71	19	38	<0.001	0.001
41–50	51–60	46	38	0.204	1.000
41–50	61–70	46	30	0.065	0.908
41–50	≥71	46	38	<0.001	<0.001
51–60	61–70	38	30	0.124	1.000
51–60	≥71	38	38	<0.001	0.004
61–70	≥71	30	38	<0.001	0.010

Note: Full genotype distributions; conditional Monte Carlo p values. Holm correction across all 21 age-group pairs.

Table S1b. Genotype-specific comparisons by sex

Genotype	Male, n	Female, n	Male, %	Female, %	Raw Fisher p	Holm-adjusted p
GT1a	16	3	18%	3%	0.001	0.009
GT1b	38	65	42%	66%	0.001	0.009
GT1 (non-1a/1b)	2	4	2%	4%	0.684	1.000
GT2	0	1	0%	1%	1.000	1.000
GT3	22	14	24%	14%	0.095	0.572
GT4	5	6	6%	6%	1.000	1.000
GT5	1	0	1%	0%	0.479	1.000
Mixed	6	5	7%	5%	0.760	1.000

Note: Two-sided Fisher exact tests; each target genotype versus all other results. Holm correction across eight tests. Percentages use n = 90 (male) and n = 98 (female) and are displayed as whole numbers.

Table S1c. Pairwise comparisons between study years

Group 1	Group 2	n1	n2	Raw p (MC)	Holm-adjusted p
2019	2020	19	76	0.019	0.249
2019	2021	19	27	<0.001	0.007
2019	2022	19	27	0.037	0.408
2019	2023	19	24	0.013	0.178
2019	2024	19	15	0.033	0.398
2020	2021	76	27	0.762	1.000
2020	2022	76	27	0.974	1.000
2020	2023	76	24	0.458	1.000
2020	2024	76	15	0.934	1.000
2021	2022	27	27	0.385	1.000
2021	2023	27	24	0.059	0.586
2021	2024	27	15	0.538	1.000
2022	2023	27	24	0.411	1.000
2022	2024	27	15	0.795	1.000
2023	2024	24	15	0.529	1.000

Note: Full genotype distributions; conditional Monte Carlo p values. Holm correction across all 15 year-pair comparisons; exploratory analysis.

Supplementary Table S2. Genotype-specific temporal trend and logistic regression analyses (N = 188)

Genotype	Events	C-A Z	C-A p	C-A Holm p	OR/year	95% CI lower	95% CI upper	Wald p	Wald Holm p
GT1a	19	-0.427	0.669	1.000	0.931	0.669	1.295	0.670	1.000
GT1b	103	1.460	0.144	0.721	1.158	0.950	1.412	0.146	0.730
GT1 (non-1a/1b)	6	-2.864	0.004	0.025	0.092	0.017	0.491	0.005	0.031
GT3	36	0.860	0.390	1.000	1.111	0.873	1.414	0.391	1.000
GT4	11	-0.916	0.360	1.000	0.809	0.511	1.280	0.364	1.000
Mixed	11	-0.495	0.621	1.000	0.897	0.581	1.383	0.622	1.000

Note: C-A: two-sided Cochran–Armitage score test. Annual ORs and nominal 95% Wald CIs; continuous year, no categorical reference year. Each target genotype was coded as 1 and all other genotype categories as 0. GT2 and GT5 were not modeled separately because each was detected in only one patient; both patients were retained in the annual denominators and comparison groups. Holm adjustment was applied across the six genotype categories separately for the C-A and Wald tests.
